# Counterparts

**Published:** 1887-06-25

**Authors:** 


### Counterparts.

By a Student of Eastern Philosophies.

*(Continued from p. 197, vol. ii.)*

Chapter III.

"I see you are inclined to be sceptical, Miss Edith."

Since the memorable day on which Archie had thought fit to visit Westborough, this was the first time that Edith had found herself alone with Paul. As if in answer to her wish, an opportunity for discussion had unexpectedly presented itself, for Miss Charteris was out at a missionary meeting, and Edith knew she would have Paul to herself for the next two hours. A few leading questions had soon set him talking on the subject which so roused her interest.

"You are sceptical just now," Paul was saying, "but one day you will be convinced of these things. You do not know yet that you are a mesmeric sensitive."

"I am afraid I am too self-willed to be anything of the kind," said Edith.

"Temperament has more to do with it than will," remarked Paul.

"But how do you know, without trying, that I am a mesmeric sensitive, as you call it?" asked Edith.

"By the magnetic *rapport* which exists between you and myself."

"I do not feel any," said Edith, thoughtlessly.

"Is that true?" asked Paul, gravely, turning his eyes full upon her.

Edith made no answer. She was put to confusion by his penetrating glance.

"Of course," said Paul slowly, "I know you do not feel the *rapport* so strongly as I do; you have various counteracting influences—one especially—bearing upon you from without. These it would not be difficult to overthrow, by the use of another and more powerful influence; but where, as in your case, the health is so good, and the balance between body, soul, and spirit so nearly perfect, it would be wrong, in my opinion, to risk the introduction of a disturbing element, by increasing the bias which you already have towards the spiritual side. Such changes should come naturally and spontaneously from within—they should not be forced."

"Do you mean that my health would suffer if I became more spiritual?"

"Yes, if the change were too sudden. The body requires to become inured to the spiritualising process, otherwise it will be consumed by the rapidly expanding soul."

"Good gracious!" said Edith faintly.

"The magnetic state helps the tendency towards spiritualisation. All the inclinations of a magnetised patient are towards goodness and morality, unless an impulse to evil is suggested by the operator. Now, by throwing you into a magnetic sleep, I could——"

"But I won't be thrown into a magnetic sleep," exclaimed Edith with great decision, and some alarm. "I should hate to be mesmerised."

"You are quite right," said Paul, quietly; "it is dangerous to tamper wantonly with the great forces of nature."

"Then why do you do it," demanded Edith—"if mesmerism *is* a great force of nature?" she added.

"I am not aware of ever doing so."

"But Archie—Mr. Bannerman said you were supposed to cure people of all manners of ailments by mesmerising them."

"And do you call that tampering wantonly with nature?" asked Paul, waxing warm, perhaps at the mention of Archie's name. "If I have effected a few cures by the faculty which in a slight degree I possess, what have I done but put to its proper use one of the best of nature's gifts to her sons? Oh, you Europeans! Britons especially," continued Paul, forgetting that he was a Briton himself, and beginning in his excitement to walk hurriedly up and down the room, while his eyes blazed forth wrath and scorn. "Short-sighted race! wise in your own conceit! who despise the teachings of your forefathers, and fancy that you have already fathomed the universe in all its immensity! who believe only in what you see with your bodily eyes, or can logically deduce from that seen by your bodily eyes! who have banished faith, spirit, God, from your miserable little world of self-existent matter! It is no wonder that of the great healing power of nature you have lost all but the instinct of self-preservation!"

Here Paul brought his apostrophe to a close as abruptly as he had commenced it, and, to Edith's great relief, came and sat down beside her in a perfectly sane and rational manner, and took up his teacup as quietly as if no sceptical race of Europeans had ever been born to disturb his equanimity.

"I suppose you have heard of the *vis medicatrix naturæ*, Miss Edith," he remarked.

"Yes," answered Edith, devoutly hoping he would not make inquiries as to the extent of her knowledge.

"You know," continued Paul, as he stirred his tea with a meditative air, "you know that in every organism there are two great principles, one of life, the other of destruction, and that death ensues only when the vital force is too feeble to exist or repair the ravages caused by the destructive force. This principle of life, the *vis medicatrix naturæ* of the doctors, without which doctors can do nothing, is generated, as you know, in the brain and other nerve-centres, and distributed by means of the nerves to the various parts of the body, all the movements of which depend upon its due circulation."

"It sounds like a lecture on physiology," thought Edith; but she was profoundly interested, and said "yes?" eagerly enough, when Paul made a pause and looked at her.

"Mesmerists possess this nerve-force in what is now considered an abnormal degree. Not only have they a vast supply for themselves and the preservation of their own health, but by the strength of their will they can project it from their own bodies into the bodies of others."

"Oh!" exclaimed Edith, looking as if this taxed her credulity too much.

"Do you find that too strange to be true? I tell you, the wonders of nature far transcend the subtlety of either our senses or our intellect. You must remember also that nerve-force is not a fluid—though it is sometimes called so for convenience—but an imponderable, which is able to pass through dense substances as easily as light through glass, and which, for its transmission from one organism to another, requires but three things—health, faith, and will. When the world was young, men possessed an almost unlimited store of this all-powerful nervous energy, hence the great age to which the patriarchs attained. Sin, unbelief, and neglect have done their utmost to disperse and destroy a power which is the natural heritage of all mankind, and were it not for the care with which it is fostered and cultivated by a few of the initiated among certain religious sects in the East, it would be lost entirely, or else appear, as it does in the Western world, in an impaired and fragmentary condition, in only a few isolated individuals. Strange that the religion of this sceptical Western world should have been founded by one in whom this faculty was so wonderfully developed! Where is now the faith by which His immediate followers were so inspired, that they also were enabled to do works almost as great as their Master? I feel assured that there are no limits to the virtue by which Christ worked, and that He meant more than a hyperbolic figure of speech when He said that by faith we could remove mountains."

Paul relapsed into silence, and his eyes took a dreamy look. Perhaps he was moving mountains in imagination.

Edith was much impressed, but during the pause which followed, a good many of Archie's wise saws occurred to her mind, and somewhat disturbed the influence which Paul was gaining over her.

"Don't you think," she said, timidly, "that imagination has a good deal to do with many mesmeric cures?"

"Undoubtedly," answered Paul readily; "there is a great deal of truth in the saying that imagination can kill or cure. A patient is much more likely to recover under the influence of hope than of dull, unnerving despair. Anything that helps the flow of the nervous current is useful."

"I should like to see you work some cures," remarked Edith, thoughtfully.

"Shall I come with you some day when you are making a round of visits to the poor and sick of the town, and convince you?"

Edith looked surprised and confused. She had had no idea that Paul knew anything of her visits to poor people.

"Why should you find it so hard to believe in the all-pervading vital force?" asked Paul; "is it not one form only of that occult medium which binds the units of this vast universe together?"

"Gravitation do you mean?"

"Call it what you will—gravitation, electricity, magnetism, molecular force, or chemical affinity—it is the regulating force of all creation, this occult interpenetration, whereby each separate existence is related to others, no matter how distant, in exact proportion to the amount of medium each contains. Why should man—the highest of nature's works, as far as we yet know, and the one most amenable to subtle influences—why should he be without this undeniable, though undemonstrable force? What is sympathy with his fellow-creatures? what is mutual attraction? what is reciprocity of thought and feeling, but one of the many modes by which this subtle force works? What is—love?"

Paul had unconsciously laid his hand on Edith's, and was gazing earnestly into her eyes. The girl did not withdraw her hand; she was awed, fascinated. His deep, musical voice filled her with a new and unutterable delight; the touch of his fingers thrilled her almost painfully. As he ceased speaking everything faded from her sight but his great, luminous eyes, "dark with excess of bright," which seemed to pierce with their rays the very depths of her soul, and with a sense of half-pleasurable helplessness she became aware that she was being mesmerised, and had already succumbed too far to the influence to be able to resist it now.

Suddenly the feeling of restraint was lifted from her. Paul had withdrawn his hand and turned away his eyes. In his eagerness to convince her of the truth of his arguments, he had unconsciously been exerting his extraordinary magnetic faculty, and it was the girl's rapt face which first apprised him of the fact. A few more seconds would have sufficed to throw her into a deep sleep, but Paul was true to his principles, and honourably resisted the temptation to exercise his power over the woman he loved. With a half-regretful sigh Edith returned to the world of everyday things.

There was a short silence. Paul gazed out of the window at the sea, while Edith took furtive glances at his sharply-cut profile. A struggle was going on in his mind, but the only signs of it in his face were a slightly increased pallor, a deepening of the lines round his mouth, a darker glow in his eyes. At last he spoke. Feverishly, passionately, almost fiercely, the words which he had in vain striven to keep back fell from his lips.

"Why should I hide it from you? Is it my fault that I met you too late? I have never sought your love, much as I desire it, for you have plighted your faith to another man, and the destiny of both of us is marred. Lord! for what sin in this, or in past ages have I been thus punished? Heaven grant that we may be divided in this life only, that we may not be doomed to wander alone through the terrible Eternity, each seeking, seeking vainly, for his counterpart, his twin-soul, without which there is no rest—no real happiness—no perfection! Edith, Edith!" he cried, bending over her with a look of infinite love and pity, "I pray that you may be happy with the husband of your choice; but oh! remember that this life is not all. Keep yourself unspotted from the world in which you must mix; guard against downward dragging influences; spiritualise yourself by meditation on holy things; pray unceasingly."

His voice broke, and he turned away abruptly.

"I am going," he said presently, in an altered tone; "forgive me if I have forgotten myself too far. I shall not trouble you again with my vain love, but I cannot regret having told you of it: you had a right to know. If ever you are in need of help, call me, and I shall surely come."

He left the room, and if he heard a faint voice uttering his name, he did not answer. Edith sank back in the chair from which she had started as the door closed behind him, and covered her face with her hands.

(*To be continued.*)

### MERCY'S FREE PORT.

A hospital is one of the free ports of corporal mercy.—Ostrich.

